# Exogenous Application of Gallic Acid Induces the Direct Defense of Tea Plant Against *Ectropis obliqua* Caterpillars

**DOI:** 10.3389/fpls.2022.833489

**Published:** 2022-02-08

**Authors:** Xin Zhang, Wei Ran, Xiwang Li, Jin Zhang, Meng Ye, Songbo Lin, Miaomiao Liu, Xiaoling Sun

**Affiliations:** ^1^Tea Research Institute, Chinese Academy of Agricultural Sciences, Hangzhou, China; ^2^Key Laboratory of Tea Biology and Resources Utilization, Ministry of Agriculture, Hangzhou, China

**Keywords:** induced defense, gallic acid, jasmonic acid, flavonoids, tea geometrid larvae

## Abstract

Gallic acid (GA), an important polyphenolic compound in the plant, is a well-known antioxidant, antihyperglycemic, and anti-lipid peroxidative agent. Recently, GA treatment exhibited ameliorative effects on plants in response to some abiotic stresses. However, the elicitation effect of GA on plant defense against herbivorous insects has not yet been reported. In this study, we found that the exogenous application of GA induced the direct defense of tea plant (*Camellia sinensis*) against tea geometrid (*Ectropis obliqua*) larvae, through activating jasmonic acid (JA) signaling and phenylpropanoid pathways. These signaling cascades resulted in the efficient induction of several defensive compounds. Among them, astragalin, naringenin, and epigallocatechin-3-gallate were the three of the most active anti-feeding compounds. However, the exogenous GA treatment did not affect the preference of *E. obliqua* female moths and larval parasitoid *Apanteles* sp. Our study suggests that GA may serve as an elicitor that triggers a direct defense response against tea geometrid larvae in tea plants. This study will help to deepen the understanding of the interaction between plants and phytophagous insects and also provide theoretical and technical guidance for the development of plant defense elicitors.

## Introduction

During the long course of co-evolution with insect herbivores, plants have evolved a series of induced defense mechanisms to cope with herbivore attacks (Howe and Jander, 2008; Wu and Baldwin, 2010). Induced defense responses are first activated by the damage- and herbivore-associated molecular patterns and then regulated by a complex signaling network, including Ca^2+^ influxes, kinase cascades, reactive oxygen species, jasmonic acid (JA), salicylic acid (SA), ethylene (ET), and hydrogen peroxide (H_2_O_2_). The network finally reshapes the transcriptomes, proteomes, and metabolomes, resulting in the production of defensive compounds, such as secondary metabolites, protein inhibitors, and herbivore-induced plant volatiles (HIPVs), which in turn increase the direct or indirect resistance of plants to herbivores (Wu and Baldwin, 2010; Schuman and Baldwin, 2016; Wang et al., 2020). A growing number of studies have shown that chemical elicitors can also trigger plant-induced defenses against herbivores, including naturally occurring phytohormones, plant volatiles, and synthetic substances (Moreira et al., 2012; Zhou et al., 2018; Chen et al., 2020). The mechanisms underlying the action of different kinds of elicitors are also different. For example, many active elicitors trigger defense responses by boosting hormonal signaling pathways, thereby regulating plant resistance against herbivores (Li et al., 2018; Xin et al., 2019; Chen et al., 2020), while a newly reported chemical elicitor 4-fluorophenoxyacetic acid was found to increase plant resistance by modulating the production of peroxidases, H_2_O_2_, and flavonoids, and directly triggering the formation of flavonoid polymers without affecting the canonical hormonal pathways (Wang et al., 2020). Thus, it is still necessary to identify new chemical elicitors that induce plant defenses against herbivores and reveal the underlying mechanisms, which would be beneficial for not only insights into the precise mechanisms of induced defense but also developing new pest control strategies.

Polyphenols are secondary metabolites widely distributed in plants, and many of them are known to play important biological roles, such as resistance factors against either microbes or herbivores (Chen, 2008; War et al., 2012; Schuman and Baldwin, 2016). Furthermore, certain polyphenol compounds have been found to act as stimulators of plant stress responses in addition to their direct antibiosis action (Gozzo and Faoro, 2013; Kamatham et al., 2016; Qiu et al., 2017). Gallic acid (GA) is an important polyphenol compound in many plants, such as the tea plant (*Camellia sinensis*). GA not only has antimicrobial and insecticidal activities but also acts as a critical contributor to tea taste (Zeng et al., 2020; Zhou et al., 2020). Recently, several researchers reported that GA exhibited its regulatory role in inducing abiotic stress tolerance of plants. For example, GA was found to enhance the defense state of the rice (*Oryza sativa*) plant by promoting the expression of genes and accumulation of phenols, flavonoids, and callose (Singh et al., 2017). GA was found to increase antioxidant activities in soybean (*Glycine max*) under cold stress *via* enhancing activities of catalase and peroxidase, but declining total ascorbate and glutathione (Ozfidan-Konakci et al., 2019), while a contrary phenomenon was reported in tomato (*Solanum lycopersicum*) that GA application protected tomato callus cells from excessive boron stress by increasing the content of ascorbate and flavonoids (Farghaly et al., 2021). The exogenous application of GA in maize (*Zea mays*) seedlings was reported to have positive effects on stress parameters and lowered the impact of oxidative stress caused by copper exposure (Yetissin and Kurt, 2020). Moreover, GA derivatives also showed plant immunity inducer properties in tobacco (*Nicotiana tabacum*) by regulating the signaling pathway leading to defense reactions (Goupil et al., 2017, 2020). However, the steered effect of GA on the resistance of plants against herbivores remains largely unknown.

The tea plant is an economically important woody species, as its young leaves are the raw material for tea processing (Zeng et al., 2020). However, the tea geometrid (*Ectropis obliqua*), one of the most common herbivores in tea plantations, always causes serious damage to tea production, reducing the quality of tea (Jiang et al., 2019; Zhang J. et al., 2020). Chemical insecticides are commonly used for controlling tea geometrid, but they are harmful to both the environment and human health (Xin et al., 2016). Although the chemical ecology investigations of the tea plant lag far behind rice, maize, and other herbaceous plants, the current development still revealed that JA, ET, auxin, and other signaling pathways were involved in induced defense response in tea plants against tea geometrid (Wang et al., 2015, 2016; Xin et al., 2017; Zhang J. et al., 2020; Zhang X. et al., 2020; Li et al., 2021). Therefore, exploiting chemical elicitors, especially natural molecules, is an efficient and feasible strategy to increase the defense resistance of tea plants to herbivores. In this study, we found that the exogenous application of GA enhanced the direct defense of tea plants to tea geometrid caterpillars, upregulated the expression levels of defensive genes related to JA singling and phenylpropanoid pathways, and finally resulted in an efficient induction of defensive compounds, which will provide theoretical and technical guidance for the development of plant defense elicitors.

## Materials and Methods

### Plant and Insects

*Camellia sinensis* cv. Longjing 43 was used in this study. Two-year-old seedlings were planted individually in plastic pots (14 cm diameter × 15 cm high) and kept in a greenhouse (26 ± 2°C, 12/12 h photoperiod, 65 ± 5% relative humidity). Each plant was irrigated once every other day and fertilized monthly with compound fertilizer. The tea plants used for the experiment were in a uniform appearance.

*Ectropis obliqua* larvae were originally obtained from the tea plantation of Tea Research Institute, Chinese Academy of Agricultural Sciences, Hangzhou, China, and reared with fresh tea shoots of Longjing 43 in net cages (75 cm × 75 cm × 75 cm). All the larvae were kept in insectary with 26 ± 2°C, 12/12 h photoperiod, and 70–80% RH. After one generation, caterpillars were used for the experiments. Pupae were distinguished according to their morphological characteristics (Miller et al., 1982) and kept in different cages separately. After eclosion, one newly emerged female moth and two male moths were paired in plastic containers (12 cm high × 7 cm diameter) for 1 day to obtain fully mated female moths. A diluted honey aqueous solution (10%) was provided as a food source. Virgin female moths 1 day after eclosion and mated female moths that never laid eggs were used for Y-tube olfactometer bioassay.

The cocoons of *Apanteles* sp., an important larval parasitoid of tea geometrid, were collected from the tea plantation of Tea Research Institute, Chinese Academy of Agricultural Sciences, Hangzhou, China, and reared on *E. obliqua* larvae. Wasps were used for the experiments over one generation. A diluted honey aqueous solution (10%) was provided as a food source. Wasps were separated by sex according to morphological characteristics, 0.5-day-old male and female wasps were paired for 24 h, and only mated female wasps were used for the bioassay.

### Gallic Acid Treatment

Gallic acid (Sigma, United States) was made up to 500 mg/L with being dissolved in double-distilled water (ddH_2_O), which is physiologically correlated with the concentration of it in tea plants (Ran et al., 2018). Treated plants were uniformly sprayed with 10 ml GA solution. Control plants were sprayed with the equivalent amount of ddH_2_O. Samples for RNA extraction were harvested at 3, 12, and 48 h post-treatment; samples for catechins and caffeine analysis were harvested at 12 h post-treatment; and samples for flavonoids analysis were harvested at 24 h post-treatment. Six independent replicates were performed for each treatment and pooled into three samples for analysis.

### RNA Extraction and Real-Time Quantitative PCR Analysis

Total RNA was extracted *via* TRIzol™ kit according to the instructions of the manufacturer (TIANGEN, Beijing, China). Primer Script™ RT Reagent Kit (TaKaRa, Dalian, China) was used for synthesizing the first-strand cDNA from the total RNA. The real-time quantitative PCR (qRT-PCR) was performed on LightCycler 480 with the SYBR Green I Master (Roche Diagnostics, Mannheim, Germany). The relative expressions were calculated using the 2^–ΔΔCt^ method. *CsGAPDH* was used as the reference gene. Primers of all the measured genes were listed in Supplementary Table 1.

### Catechins and Caffeine Analysis

The analysis of catechins and caffeine was performed following the International Standards Organization (ISO) ISO 14502-2-2005 (E) procedure with minor modifications (International Organization for Standardization [ISO], 2005); 100 mg tea leaf powders were extracted by 1 ml 70% methanol. Samples were analyzed by high-performance liquid chromatography (HPLC). The separations were carried out using a Waters e2695 series HPLC system (Waters Co., Milford, MA, United States) equipped with a reversed-phase C12 column (4 μm, 250 mm × 4.6 mm i.d., Phenomenex) and a C12 guard column, and the eluate was monitored by UV spectroscopy using a diode array detector at 280 nm. Compounds were confirmed by comparison of retention time with internal standards, and the content of individual compounds was calculated by the external standard method, *via* comparing the chromatographic peaks with that of the internal standard. Standard chemicals were purchased from Sigma Chemical Company (St. Louis, MO, United States).

### Flavonoids Analysis

According to the method described by Lin et al. (2020), 200 mg leaf powders were extracted with 1 ml 80% methanol for analyzing flavonoids, and then, the mixture was vortexed for 10 min and sonicated for 5 min, for a total of three times. After centrifugation at 12,000 rpm for 30 min, the supernatant was filtered through a 0.22 μm nylon membrane filter into autosampler vials. The samples were measured by the UPLC/MS/MS analysis with the Waters ACQUITY UPLC H-Class System (Milford, MA, United States) coupled to Waters Xevo TQ-S Micro Triple-Quadrupole Mass Spectrometer. A total of 11 flavonoids, namely, astragalin, naringenin, apigenin-5-*O*-glucoside, cosmosiin, isovitexin, prunin, luteolin-7-*O*-glucoside, isoorientin, rutin, eriodictyol-7-*O*-glucoside, and isoquercitrin, were detected. The content of flavonoids was quantified using the calibration curves of corresponding standard solutions.

### Herbivore Performance Bioassay

The most apical unfolded, but not yet expanded, the leaf was designated as leaf position one, and the other leaves were numbered sequentially down the stem. The second leaves of control and GA-treated tea plants were covered by a fine-mesh sleeve and introduced with one 2-day-old caterpillar which has been starved for 8 h (*n* = 50). During feeding, if the leaf were almost half exhausted, the caterpillar would be transferred to nearby intact leaves in the same branch. Larval mass was measured 5, 9, and 13 days after the inoculation.

### Diet Assay

According to the method described by Yang et al. (2013), an artificial diet supplemented with GA, naringenin, or prunin was used for herbivore performance bioassay. Different gradients based on the physiological concentration in plants (GA with 0 and 500 mg/L; naringenin with 0, 3, and 6 μg/L; prunin with 0, 100, and 500 μg/L) were set for each treatment. Each concentration was replicated 40–50 times. At 7 and 10 days after the start of feeding, the larval mass was weighted.

### Preference Bioassay

The preference behavior of *Apanteles* sp. wasps and virgin or mated *E. obliqua* female moths were tested in a Y-tube olfactometer. *Apanteles* sp. wasps were allowed to choose between GA-treated plant and control plant. The behavioral responses of virgin or mated *E. obliqua* female moths to a pair of odors (caterpillars feeding treatment vs. GA plus caterpillars feeding treatment) were tested. The potted tea plant infested with 30 s-instar caterpillars (starved for 8 h before being used) for 24 h and then removed was regarded as caterpillars feeding treatment. The potted tea plant was pre-treated with GA for 24 h and then treated as the above was regarded as GA plus caterpillars feeding treatment. The parasitoids or moths that did not choose within 5 min will be defined as a non-responding individual and recorded as “no choice.” The position of tubes attached to sample jars was reversed after each replication to remove directional bias. After testing four wasps or moths, the olfactometer tube was replaced. The bioassay was conducted between 15:00 pm and 18:00 pm and replicated at least thirty-two times.

### Statistical Analysis

All the statistical analyses were performed by the Statistica (Institute Inc., Cary, NC, United States). The Student’s *t*-test was used for comparing differences between the treatment and control. Effects of metabolites at different concentrations on the growth of larvae were analyzed *via* one-way ANOVA with Turkey’s honest significant difference (HSD) *post hoc* test for multiple comparisons, and the correlation between the parameters was performed by Pearson’s correlation coefficient analysis. The differences in the number of *Apanteles* sp. female adults entering each arm of the olfactometer were analyzed by Kruskal–Wallis test (χ^2^ approximation).

## Results

### Gallic Acid Treatment Enhances the Direct Resistance of Tea Plants to Tea Geometrid Larvae

**FIGURE 1:**
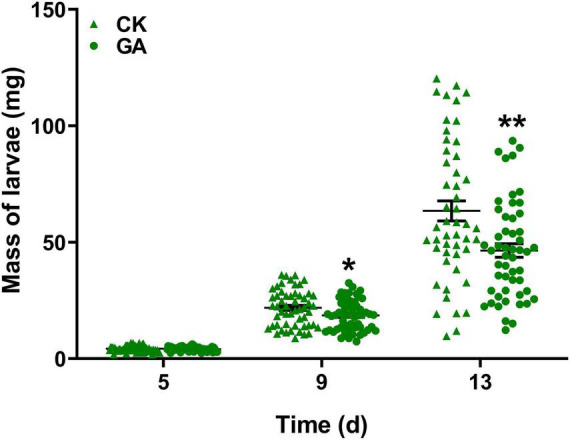
Effect of gallic acid (GA)-treated tea plants on *Ectropis obliqua* larval weight gain. Data are presented as means ± SE (*n* = 50), and asterisk indicates significant differences between GA treatment and control (*P < 0.05, **P < 0.01, Student’s *t*-test).

**FIGURE 2:**
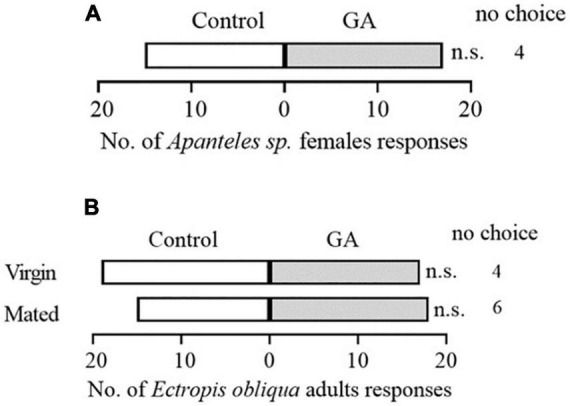
Behavioral responses of mated *Apanteles* sp. female wasps to volatiles emitted from GA-treated vs. control plant **(A)**, and mated or virgin *Ectropis obliqua* female moths to volatiles emitted from GA-treated plus caterpillars feeding vs. caterpillars feeding plants **(B)**. Numbers refer to the number of each tested insect choosing an odor source. *n* > 32 per treatment. *n.s.*, *P* > 0.05, χ^2^ test.

To explore the potential effect of exogenous application of GA on the direct resistance of tea plants, we compared the weight gain of tea geometrid fed with GA-treated and control plants. The results showed that the exogenous application of GA to tea plants significantly reduced the weight gain of larvae by 14.8 and 26.8% at 9 and 13 days, respectively, compared with control (Figure 1). Furthermore, we found that an artificial diet supplemented with GA in a similar concentration to the exogenous application did not affect the weight gain of caterpillars (Supplementary Figure 1). The data demonstrate the anti-feeding effect of GA treatment probably *via* triggering the defense responses in tea plants. However, GA treatment did not influence the preference behavior of *Apanteles* sp. (Figure 2A) and the behavioral responses of virgin or mated *E. obliqua* female moths (Figure 2B).

### Gallic Acid Treatment Upregulates the Expression Levels of Key Defensive Genes in Tea Plants

**FIGURE 3:**
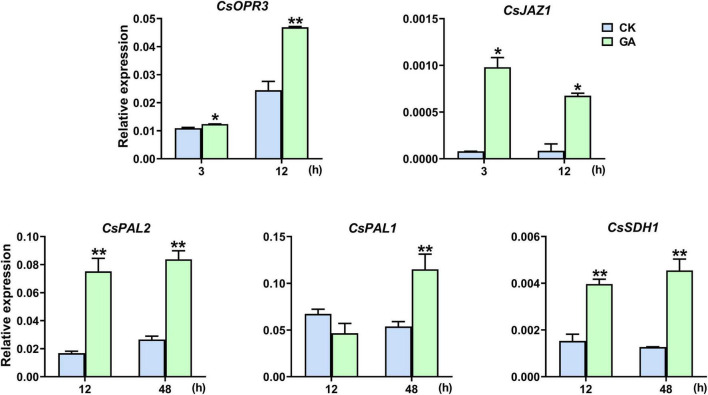
GA treatment upregulated the expression levels of genes. Data are presented as means + SE (*n* = 3), and the asterisk indicates significant differences between GA treatment and CK (*P < 0.05, **P < 0.01, Student’s *t*-test).

*CsOPR3* and *CsJAZ1* are key component genes of the JA signaling pathway, and *CsPAL1/2* and *CsSDH1* are vital synthetic genes of the phenylpropanoid pathway. GA treatment promoted the transcription of *CsOPR3*, and the level was induced to 1.9 times that in control; the level of *CsJAZ1* was induced to 12.2 and 7.9 times that in control at 3 and 12 h, respectively (Figure 3). In addition, the expression of *CsPAL1*, *CsPAL2*, and *CsSDH1* was also significantly upregulated by GA treatment, and the levels were induced to 2.1, 4.5, and 3.6 times, respectively, of that in control (Figure 3). The results suggested that the exogenous application of GA steered the synthesis and transduction of the JA and phenylpropanoid pathways.

### Gallic Acid Treatment Alters the Metabolic Profile of Tea Plants

**FIGURE 4:**
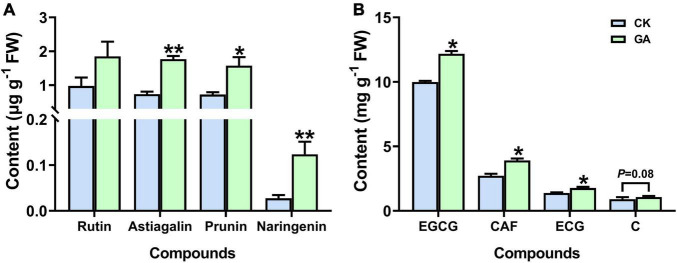
GA treatment enhanced the accumulation of metabolites. **(A)** Flavonoids. **(B)** Caffeine and catechins. Data are presented as means + SE (*n* = 3), and the asterisk indicates significant differences between GA-treated and control plant (*P < 0.05, **P < 0.01, Student’s *t*-test).

Astragalin, prunin, and naringenin (3 out of the 10 detected flavonoids) accumulated significantly in the GA treatment, which was increased by 2.4, 2.2, and 4.5-folds higher than those in control (Figure 4A and Supplementary Figure 2). In addition, significant induction in the content of caffeine and galloylated catechins was also noticed in the GA-treated plants. The concentration of caffeine was significantly increased by 43%; epicatechin-3-gallate (ECG) and epigallocatechin-3-gallate (EGCG) were significantly increased by 22 and 27%, respectively (Figure 4B). Thus, the exogenous application of GA altered the metabolic profile of tea plants by regulating the synthesis of metabolic compounds related to flavonoids, caffeine, and catechins. Moreover, the effects of prunin and naringenin were selected for further verification.

### Effects of Prunin and Naringenin on the Performance of Tea Geometrid

**FIGURE 5:**
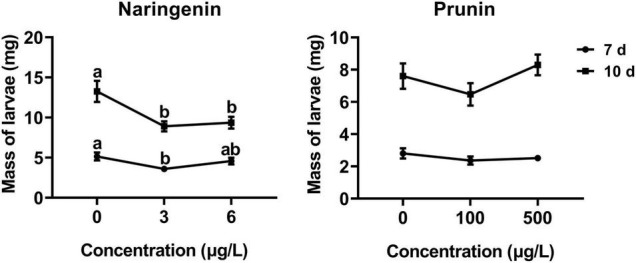
Effects of naringenin and prunin on the weight gain of tea geometrid larvae. Data are presented as means ± SE (*n* = 40), and different letters indicate significant differences among treatments [*P* < 0.05, Turkey’s honest significant difference (HSD) *post hoc* test].

The results of this study showed that the addition of naringenin significantly decreased the larval weight, with a reduction of about 30% compared with control, except the 7 days at the concentration of 6 μg/L. In addition, there was a negative correlation between larval weight and naringenin concentration at 10 days (*r* = −0.280, *P* = 0.005). Compared with control, the addition of prunin slightly, but not significantly, affected the growth ratio of tea geometrid (Figure 5).

## Discussion

Plant-induced defense against herbivore attacks can be deployed as an important strategy in pest management. So far, a large number of studies have shown that chemical elicitors and small molecular compounds can increase the resistance of plants against insects by inducing defensive responses (Xin et al., 2016; Lin et al., 2020; Wang et al., 2020). In this study, we found that the exogenous application of GA induced the direct resistance of tea plants to tea geometrid larvae. The expression levels of genes related to JA and phenylpropanoid pathways were upregulated, and the accumulation of caffeine, galloylated catechins, astragalin, prunin, and naringenin was enhanced. Furthermore, astragalin, naringenin, and EGCG were proved to be the three most active anti-feeding compounds. However, GA treatment did not affect the preference of *E. obliqua* female moths and parasitoid *Apanteles* sp. These results provide shreds of evidence for GA, a natural compound in the plant, acting as an elicitor that increases plant resistance to herbivore larvae but did not affect volatile organic compounds-mediated defenses to the insect and its natural enemies.

Jasmonic acid and phenylpropanoid pathways play key roles in induced defensive responses and have been reported in several plants (Howe and Jander, 2008; Lou et al., 2015; Schuman and Baldwin, 2016). Transcriptome analysis revealed that genes related to JA signaling and phenylpropanoid biosynthesis are involved in inducible defenses responses in tea plants against tea geometrid (Wang et al., 2015, 2016). Previously, 12-oxophytodienoate reductase 3 (OPR3) was reported to act as a key enzyme in the biosynthesis of JA and its mutant plant changed JA-induced resistance to insects (Browse, 2009), while JAZ1 is an important component of JA signaling transduction, which has been identified as an early JA-responsive gene (Staswick, 2008; Oh et al., 2012). Our previous works have demonstrated that both *CsOPR3* and *CsJAZ1* in tea plants were responsive to JA and tea geometrid attacks (Xin et al., 2017; Zhang X. et al., 2020). In this study, we found that the expression levels of JA pathway-related genes, *CsOPR3* and *CsJAZ1*, and phenylpropanoid pathway genes, *CsPAL1/2* and *CsSDH*, were upregulated by GA treatment. In line with that GA has been reported to activate the expression of phenylpropanoid pathway genes, *OsPAL* and *OsCHS*, and methyl jasmonate-regulated gene, *OsWRKY71*, in rice (Singh et al., 2017). Moreover, the upregulation of transcription levels of genes related to JA signaling and phenylpropanoid pathways in GA-treated plants may be correlated with the accumulation of defensive compounds, which in turn regulate antioxidants and alleviate reactive oxygen generation in rice (Singh et al., 2017). The exogenous application of GA in tea plants also elicited subsequent responses with the production of defensive secondary metabolites. Our results suggested that GA may activate JA signaling and phenylpropanoid pathways to modulate tea plant resistance.

Plants develop a strategy to accumulate a variety of metabolites against herbivorous insects, including flavonoids, caffeine, and nitrogen and sulfur-containing compounds (Treutter, 2006; Chen, 2008; Mazid et al., 2011). Hitherto, the defensive role of secondary metabolites in plant-insect interactions has been widely elucidated (Mazid et al., 2011; Gols, 2014). They may act as feeding deterrents, digestibility reducers, and toxins of herbivores, and their accumulation is often enhanced by insect infestation (Mazid et al., 2011). For example, naringenin was reported to inhibit the larval growth and development of the common cutworm *Spodoptera litura* (Stec et al., 2020). Transgenic tobacco with higher content of caffeine exhibited a relatively high resistance to herbivores (Ashihara et al., 2008). In tea plants, caffeine was found to inhibit the oviposition of *Xyleborus fornicatus* and protect tender tissues from the damage of beetles (Hewavitharanage et al., 1999). Our group also found that astragalin negatively affected the growth rate of tea geometrid, and the anti-feeding effect was intensified by feeding time (Lin et al., 2022, accepted). JA was involved in the induction of EGCG, and there was a negative correlation between the level of EGCG in the diet and tea geometrid larval mass (Li et al., 2021). In this study, significant induction of flavonoid compounds by GA treatment was noticed, especially naringenin, astragalin, and EGCG. In addition, an artificial diet supplemented with naringenin significantly reduced the larval growth rate of tea geometrid. Consistent with previous results in rice and tomato plants, the exogenous treatment of GA promoted the total flavonoids content (Singh et al., 2017; Farghaly et al., 2021). Our study provided further evidence on which flavonoids were regulated by GA. Thus, the induction of naringenin, astragalin, EGCG, and caffeine might be the reason for the larval growth reduction in GA-treated plants. Although the increment of caffeine and flavonoids in GA-treated plants may attribute to the enhanced resistance of tea plants, other metabolites may also function as defense compounds against tea geometrid, and the underlying mechanisms still need deeper investigation in the future.

## Conclusion

The exogenous application of GA induced the direct defense of tea plants against tea geometrid larvae, through activating the JA signaling and phenylpropanoid pathways. Astragalin, naringenin, and EGCG were the three most active anti-feeding compounds, whose accumulation was enhanced by the exogenous application of GA in tea plants. However, the exogenous GA treatment did not affect the preference of *E. obliqua* female moths and larval parasitoid *Apanteles* sp. Our study suggests that GA may serve as an elicitor that triggers the direct defense response of tea plants against tea geometrid larvae.

## Data Availability Statement

The raw data supporting the conclusions of this article will be made available by the authors, without undue reservation.

## Author Contributions

XZ, WR, and XS: conceptualization. XZ, SL, and ML: experiments and data analysis. XZ and XL: materials. XZ: writing – original draft preparation. JZ, MY, and XS: writing – review and suggestion. All authors read and agreed to the published version of the manuscript.

## Conflict of Interest

The authors declare that the research was conducted in the absence of any commercial or financial relationships that could be construed as a potential conflict of interest.

## Publisher’s Note

All claims expressed in this article are solely those of the authors and do not necessarily represent those of their affiliated organizations, or those of the publisher, the editors and the reviewers. Any product that may be evaluated in this article, or claim that may be made by its manufacturer, is not guaranteed or endorsed by the publisher.
