# Supplementary material for: Exogenous Application of Gallic Acid Induces the Direct Defense of Tea Plant Against Ectropis obliqua Caterpillars
Source: Front Plant Sci. 2022 Feb 8;13:833489. doi: 10.3389/fpls.2022.833489 (PMC8861190; doi:10.3389/fpls.2022.833489)
Supplement: Supplementary file 1 [file Data_Sheet_1.docx]

Exogenous application of gallic acid induces the direct defense of tea plant to *Ectropis obliqua* larvae

Xin Zhang^1,2^, Wei Ran^1,2^, Xiwang Li^1,2^, Jin Zhang^1,2^, Meng Ye ^1,2^, Miaomiao Liu^1,2^, Xiaoling Sun^1,2*^

^1^ Tea Research Institute, Chinese Academy of Agricultural Sciences, No. 9 South Meiling Road, Hangzhou, Zhejiang, China

^2^ Key Laboratory of Tea Biology and Resources Utilization, Ministry of Agriculture, No. 9 South Meiling Road, Hangzhou, Zhejiang, China

*** Correspondence:**Corresponding Author
xlsun@tricaas.com/xlsun1974@163.com

**Supplementary Table S1.** Primers for qRT-PCR analysis.

| Primers | Primer sequence (5'–3') |
| --- | --- |
| *CsOPR3-QF* | CTCTCTCACAGGGTGGTGCT |
| *CsOPR3-QR* | CCAGGGCAATGTGGAAACCC |
| *CsJAZ1-QF* | CGGCCATAGACACTCCTCCA |
| *CsJAZ1-QR* | TGGCATGCCGAGACTGAGAT |
| *CsPAL2-QF* | CCAATTCCTTGCCAATCCTGTAAC |
| *CsPAL2-QR* | CAACTGCCTCGGCTGTCTTTCT |
| *CsSDH1-QF* | TTGTGCTCCATTGATGAGTC |
| *CsSDH1-QR* | GAGGAGGATTTGGAGGTCTC |
| *GAPDH-QF* | GACTGGAGAGGTGGAAGAGC |
| *GAPDH-QR* | AGCCATTCCAGTCAATTTCC |


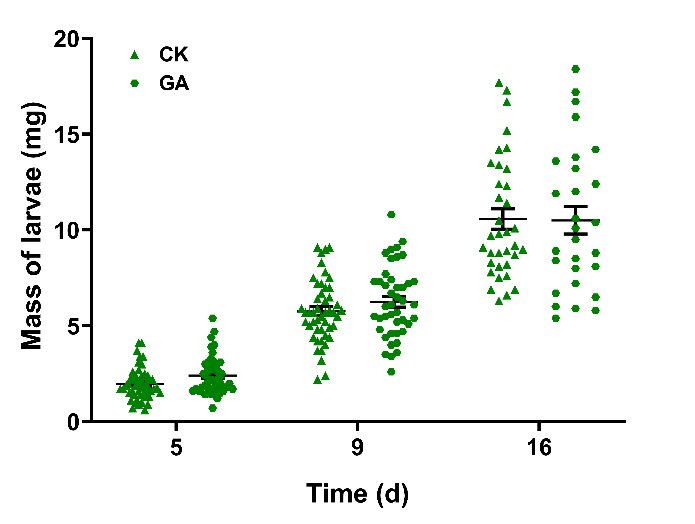


**Supplementary Figure 1.** Effect of GA on the weight gain of tea geometrid larvae in artificial diet assay. Data are presented as means ± SE (*n* = 40, Student’s *t* test).


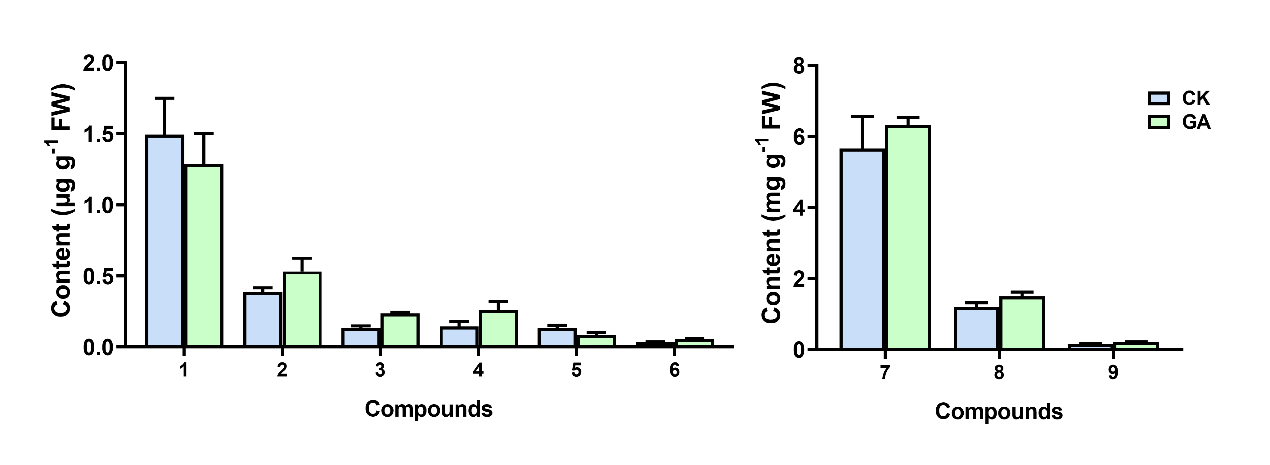


**Supplementary Figure 2.** Effects of GA treatment on the accumulation of metabolites in tea plant. 1. Isovitexin; 2. Cosnosiin; 3. Luteolin-7-o-glucoside; 4. Isoquerctrin; 5. Isoorientin; 6. Eriodictuol-7-o-glucoside; 7. epigallocatechin; 8. epicatechin; 9. gallocatechin. The data are presented as means + SE (*n* = 3, Student’s *t* test).
